# Medication errors in a cohort of pediatric patients with acute lymphoblastic leukemia on remission induction therapy in a tertiary care hospital in Mexico

**DOI:** 10.1002/cam4.2438

**Published:** 2019-08-24

**Authors:** Edmundo Vázquez‐Cornejo, Olga Morales‐Ríos, Luis E. Juárez‐Villegas, Erika J. Islas Ortega, Felipe Vázquez‐Estupiñán, Juan Garduño‐Espinosa

**Affiliations:** ^1^ Post‐degree in Medical, Dentistry and Health Sciences Universidad Nacional Autónoma de México Ciudad de México Mexico; ^2^ Evidence‐based Medicine Unit Hospital Infantil de México Federico Gómez Ciudad de México Mexico; ^3^ Department of Clinical Research Hospital Infantil de México Federico Gómez Ciudad de México Mexico; ^4^ Department of Oncology Hospital Infantil de México Federico Gómez Ciudad de México Mexico; ^5^ Department of Pharmaceutical Services Hospital Infantil de México Federico Gómez Ciudad de México Mexico; ^6^ Hospital Ángeles Clínica Londres Ciudad de México Mexico; ^7^ Directorate of Research Hospital Infantil de México Federico Gómez Ciudad de México Mexico

**Keywords:** acute lymphoblastic leukemia, adverse drug events, developing countries, medication errors, patient safety, quality of care

## Abstract

**Introduction:**

Medication errors (MEs) are the main type of preventable adverse events in medical care, as well as safety indicators in the medication processes. Advances in the quality of care in pediatric acute lymphoblastic leukemia (ALL) have enabled to improve clinical outcomes. However, ME epidemiology in pediatric oncology is still incipient in developing countries. In view of this, the objectives of this study were to estimate the incidence of MEs, determine their types and consequences, as well as their preventability in the induction treatment of children with ALL at Hospital Infantil de Mexico Federico Gómez.

**Methods:**

We reviewed the remission‐induction chemotherapy records of children with ALL between January 2015 and December 2017. A two‐phase review was carried out for ME identification and verification. The consequences of errors were determined by agreement between reviewers.

**Results:**

We reviewed 1762 chemotherapy orders involving 181 children. MEs were observed in 16.9% of orders and in 57.5% of patients. Prescription errors were the most common (93.3%), with wrong dose errors (90.2%) being predominant. Only 3.7% of wrong dose errors were intercepted, while 12.2% of the children experienced adverse drug events (ADEs) preceded by some wrong dose error.

**Conclusions:**

MEs were common, since they occurred in 57.5% of children with ALL on induction treatment and involved 16.5% of chemotherapy orders. Only 3.7% of MEs were intercepted, while 12.2% of children had ADEs related to overdose. Measures are required to prevent calculation error in prescriptions, as well as training of the nursing staff to intercept MEs.

## Graphical abstract

This study is a retrolective cohort whose aim is estimates the incidence and consequences of medication errors in children with acute lymphoblastic leukemia on remission induction therapy in a tertiary care hospital in Mexico. Medication errors were observed in 16.9% of orders and in 57.5% of patients, while 12.2% of the children experienced adverse drug events preceded by some wrong dose error. Preventive measures are required to build a safer medication process.

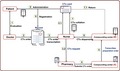

## 1 INTRODUCTION

Medication errors (MEs) are the main type of preventable adverse event in health care,^1^, ^2^ and are considered an indicator of poor quality and safety in the processes of patient treatment.^3^ MEs are defined as any preventable event that may cause or lead to inappropriate medication use or patient harm while the medication is under the control of the healthcare professional patient or consumer.^4^

Although MEs epidemiology in pediatric oncology has been widely described in developed countries,^5^, ^6^, ^7^, ^8^, ^9^ such information is still incipient in developing countries.^10^, ^11^ Especially in Mexico the frequency and consequences of MEs in children receiving chemotherapy have not been studied. However it has been pointed out that the study of specific aspects of the quality and safety of the care processes should produce preventive measures that improve outcomes within the hospital.^12^, ^13^, ^14^, ^15^

In recent decades advances in the quality of childhood acute lymphoblastic leukemia (ALL) processes of care have improved survival in developed countries.^16^, ^17^ At Hospital Infantil de México Federico Gómez (HIMFG) the main cause of medical care is ALL. It accounts for 39.7% of children with neoplasms in our hospital^18^ and maintains an elevated early mortality,^13^, ^19^ such as in other developing countries.^20^, ^21^

On the other hand the remission induction phase is critical for children with ALL: they receive intensive chemotherapy in a short period while the leukemia is still active and they are more susceptible to treatment‐related toxicity.^22^, ^23^ For these reasons MEs in this phase are particularly relevant.

Considering the above the purpose of this study was to estimate the incidence of MEs during the remission induction treatment of children with ALL, to determine their types and consequences as well as the preventability of such MEs within the medication system.

## 2 MATERIALS AND METHODS

### 2.1 Setting

**Figure 1:**
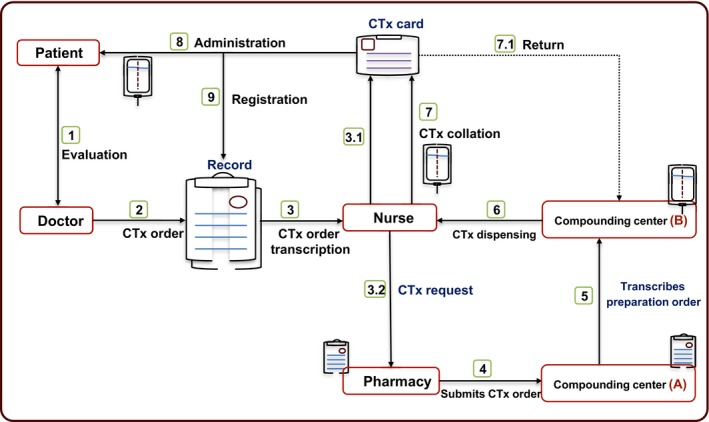
Oncology department medication system: (1) The patient is assessed by the doctor. (2) An attending or resident doctor manually writes down the chemotherapy (CTx) order in the medical record. (3) The nurse transcribes said order in two formats: (3.1) first, in the patient CTx card, and then, (3.2) in a CTx request form that is submitted to the hospital pharmacy. (4) The pharmacy assigns a file number and forwards the request to a processing station for requests to the External Compounding Center (ECC); this station is located inside the hospital (A). (5) The station manually transcribes the CTx request prepared by the nursing staff on an electronic platform and sends it to its external facilities (B), where the CTx is prepared. (6) The ECC dispenses the prepared CTx to the hospital. (7) The nurse receives the CTx, collates it with the patient card and keeps it safe. (7.1) If it is incorrect, it is returned to the ECC. (8) If the CTx is correct, it is administered to the patient. (9) The nurse registers the administration in the patient record

HIMFG is a national pediatric tertiary care institute in Mexico that has 229 beds available, and where an average of 7729 annual discharges take place, with 25.8% corresponding to cancer patients. The medication system at the oncology department comprises nine steps that start with the manual preparation of the chemotherapy order by the attending or resident doctor and conclude when the chemotherapy administration is registered by the nurse in the medical record (Figure 1). Each chemotherapy order is transcribed in four stages of the process: three by the nurse and one by the staff of the External Compounding Center (ECC) that prepares the chemotherapy outside the HIMFG. Since 2016, the ECC evaluates the stability and safety of the received chemotherapy orders and observations are reported to the doctor, who decides whether to accept them or not.

### 2.2 Remission induction therapy

At HIMFG, children with ALL receive a standard induction treatment that includes an 8‐day corticosteroid window (dexamethasone or prednisone), followed by daily administration until remission is assessed at induction day 28, four once‐weekly doses of vincristine (2 mg/m^2^), two once‐weekly doses of daunorubicin (25 mg/m^2^) on the first 2 weeks and nine doses of L‐asparaginase (10 000 IU/m^2^), as well as weekly CNS prophylactic treatment. Children younger than 1 year or weighing less than 10 kg receive a vincristine weight‐adjusted dose (0.05 mg/kg). When a patient experiences infections or toxic effects that delay the treatment for less than 2 weeks, therapy is resumed and the cycle is completed; otherwise, the treatment is restarted.^24^

### 2.3 Study design and patients

This study is a retrolective cohort from medical records of children aged between 0 and 18 years, who were newly diagnosed with ALL at HIMFG between January 2015 and December 2017. Children with ALL who did not receive induction chemotherapy treatment at HIMFG were excluded, as well as when the medical records did not document at least one induction protocol‐based chemotherapy order from prescription to administration.

### 2.4 Reliability and validity

The validity of the clinical records review was assessed in two scrutiny stages. In the first one, one of the researchers (EVC) used a form for systematic extraction of clinical records’ therapeutic information, based on a previously described method.^25^ Then, he classified the types of ME in the medication system, and adverse drug events (ADEs) during the induction period. In the second stage, in an effort to corroborate the MEs identified in the first stage, another investigator (OMR)  independently reviewed a random sample of clinical records. The reliability of the judgement to identify MEs was assessed by means of a weighted kappa test (*K*_w_). The observed agreement was very good (*K*_w_ = 0.91; 95% CI, 0.82‐0.99).

### 2.5 Follow‐up

The clinical records of children with a primary diagnosis of ALL who met the inclusion criteria were reviewed by one researcher (EVC) who extracted demographic, clinical, and therapeutic information. The extracted demographic and clinical variables were the following: gender, age (from 1 to 9.9 years vs < 1 and ≥ 10 years), white blood cell (WBC) count (< 50 × 10^9^/L vs ≥ 50 × 10^9^/L), clinical status at admission, which was defined as serious when the patient was admitted to the intensive care unit. In addition, the body mass index was transformed into z‐score standard deviations (SD), calculated with the WHO AnthroPlus tool to assess the growth of children and adolescents from 0 to 19 years of age (≥ −1 SD to ≤ +1 SD, adequate; < −1 SD, undernourishment; > +1 SD to ≤ +2 SD, overweight and > +2 SD, obesity). At diagnosis, data on immunophenotype (B, T or mixed leukemic cells), cell morphology (L1, L2 or L3) and leukemia cytogenetics (unfavorable: t[9;22] Philadelphia+, t[4;11] and t[1;19]; favorable: t[11;19], t[12;21]) were collected, as well as on central nervous system (CNS) status (no blasts, infiltrated, with CNS hemorrhage, traumatic puncture and uncertain) and risk classification (standard risk: from 1 to 9.9 years of age and WBC < 50 × 10^9^/L, and high risk: ≥ 10 years and < 1 year of age, WBC ≥ 50 × 10^9^/L)^26^ and morbidities additional to ALL.

To extract the induction phase therapeutic information, one researcher (EVC) reviewed the chemotherapy orders documented in the medical record and collected data on each prescribed chemotherapeutic drug, recording the generic name of the drug, the dosage ordered by the doctor, the calculated dose and route of administration, as well as the patient weight (kg) and body surface area (m^2^) on the date the chemotherapy order was prepared. On the other hand, information on medications was collected from the nursing administration records, with the medications name and the administered quantity, route, and dates being recorded. In addition, all ADEs documented in the record during the remission induction phase were extracted.

### 2.6 Medication error measurement

To determine the presence of ME, the American Society of Health‐System Pharmacists definitions of prescribing, transcription, and administration error were used.^27^ In the prescribing process, chemotherapy orders whose quantity of drug had differences greater than 10%^6^, ^8^, ^10^ with regard to the quantity recalculated by the reviewer, were considered wrong doses. To recalculate the quantity of drug of each chemotherapy order, the dosage indicated by the physician and patient weight or body surface area on the date of the order were used.

Deviations from the chemotherapy order prescribed by the doctor occurring in the request for chemotherapy preparation (by the nurse or the ECC) until the chemotherapy admixtures were dispensed for administration to the patient were considered transcribing errors. These deviations were assessed by comparing the ECC dispensing records, which are kept in the hospital pharmacy (EJIO), with the prescribed orders, as well as with the medication administration records registered in the patient chart by the nurse. Administration error was restricted to corticosteroid omission during hospital stay.

### 2.7 Consequences

An ADE was defined as any unintended harm related to the chemotherapy treatment,^3^, ^28^ but preceded by overdose. Intercepted prescribing errors were analyzed by the research team in order to determine the potential effects based on the patient clinical condition and the chemotherapy toxicities described in the literature.^29^ To grade the severity of ADEs and potential ADEs, the scale created by the National Coordinating Council for Medication Error Reporting and Prevention (NCC MERP) was used.^28^ The classification ranged from MEs without consequences to MEs that might have contributed to death. Finally, the degree of preventability of ME‐preceded ADEs was determined based on published medical evidence.

### 2.8 Statistical analysis

The incidence of MEs, as well as that of ADEs, was calculated based on the number of children with at least one ME for every 100 on induction treatment. In addition, the incidence of chemotherapy orders with any ME was estimated for every 100 orders documented in the study period. For both estimates, the 95% confidence interval was determined.

For the description of categorical variables, frequencies and percentages were used. The ME variables for each type were dichotomized for descriptive analysis (with and without error), both by patients and by chemotherapy order. An exploratory analysis of the presence of ME by subgroups in the cohort was carried out using the chi‐square or Fisher's exact test, as appropriate, with a two‐tailed *α*‐value of 0.05 being accepted. Statistical analyses were performed using the IBM SPSS Statistics 25 software.

### 2.9 Ethics

The study was approved by the HIMFG ethics, research, and biosafety committees with protocol number HIM 2018‐018. Patient privacy and anonymity was ensured in the generated database.

## 3 RESULTS

### 3.1 Patients

**Figure 2:**
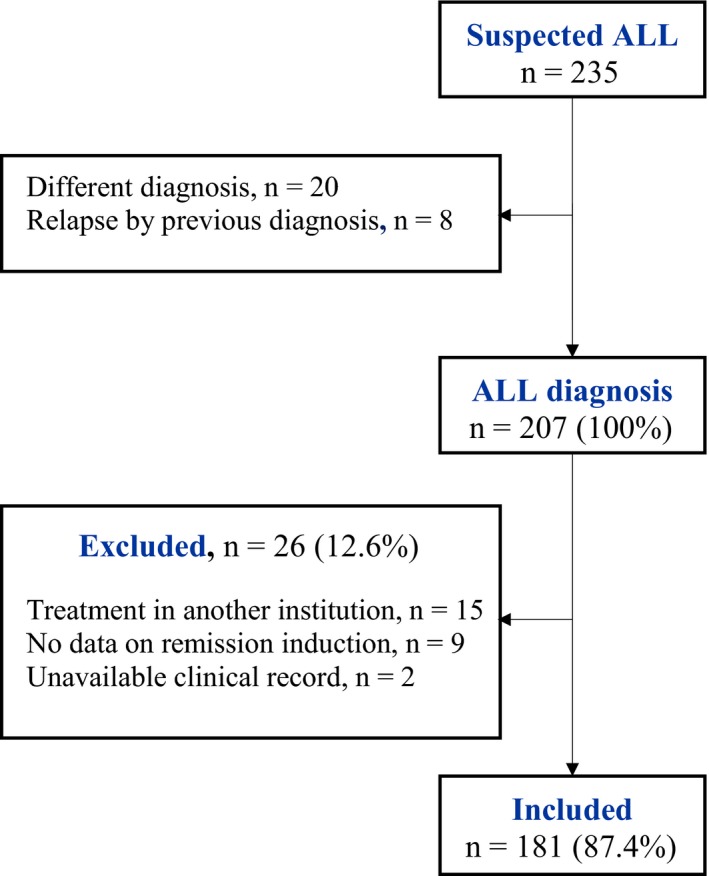
Process of clinical records selection for the cohort. ALL: acute lymphoblastic leukemia

After reviewing 205 (99.0%) clinical records of newly diagnosed cases of ALL, 181 (87.4%) were included (Figure 2). Age ranged from 17 days to 16.9 years. Patients classified at high risk (65%) predominated, 51.4% had some degree of malnutrition, and one‐fifth (18.8%) arrived in clinically serious conditions at the time of diagnosis. Induction treatment had a mean duration of 36.8 days (± 8.6 days) (Table 1).

**Table 1:** Baseline characteristics of children with acute lymphoblastic leukemia

Characteristic	n = 181
Gender—n (%)	
Males	89 (49.2)
Females	92 (50.8)
Age (years)—me (IQR)	7.3 (3.7, 11.1)
Age (years)—n (%)	
1‐9	107 (59.1)
<1 and ≥ 10	74 (40.9)
Nutritional status (*z*‐score)—n (%)	
Adequate (≥ −1.0 SD to ≤ +1.0 SD)	88 (48.6)
Undernourishment (< −1.0 SD)	38 (21.0)
Overweight (> +1.0 SD to ≤ +2 SD)	34 (18.8)
Obesity (> +2.0 SD)	21 (11.6)
Leucocytes (10^9^/L)—n (%)	
<50.0	141 (77.9)
≥50.0	40 (22.1)
Inmunophenotype—n (%)	
B	167 (92.3)
T	11 (6.1)
Mixed	3 (1.6)
Cell morphology—n (%)	
L1	42 (23.2)
L2	136 (75.1)
L3	3 (1.7)
Cytogenetics—n (%)	
Unfavorable	23 (12.7)
Favorable	11 (6.1)
CSF status at diagnosis—n (%)	
Absence of blasts	155 (85.6)
Infiltrate	14 (7.7)
CNS hemorrhage	4 (2.2)
Traumatic puncture	3 (1.7)
Uncertain	5 (2.8)
Risk classification—n (%)	
Standard	64 (35.4)
High	117 (64.6)
Clinical status at admission—n (%)	
Stable	147 (81.2)
Serious	34 (18.8)
Comorbidities—n (%)	
None	162 (89.5)
≥ 1	19 (10.5)

CNS, central nervous system; CSF, cerebrospinal fluid; IQR, interquartile range; Me, median; SD, standard deviation;

### 3.2 Medication error incidence

In this cohort, an accumulated incidence of ME of 57.5% was observed in children on treatment, while in the prepared chemotherapy orders, MEs occurred at a rate of 16.9% (Table 2). Of all MEs, 31.6% occurred with corticosteroids (26.2% with dexamethasone), followed by 25.5% with L‐asparaginase, 20.8% with vincristine and 15.8% with daunorubicin; the remaining 6.3% occurred with other drugs used in the remission induction period (Figure S1).

**Table 2:** Incidence of medication errors and adverse drug events

	Patients (n = 181)			Chemotherapy orders (n = 1762)		
Type of error	n	I^a^	95% CI	n	I^a^	95% CI
Medication error	104	57.5	50.3‐64.7	298	16.9	15.2‐18.7
Adverse drug event	22	12.2	7.4‐16.9	25	1.4	0.9‐2.0

a Incidence per 100 patients or chemotherapy orders on induction treatment.

### 3.3 Types of medication error

In the prescribing process, 278 (15.8%) chemotherapy orders had MEs, with one or more of them occurring in 89 children (49.2%) (Table 3). Wrong dose errors were the most common MEs in the course of the follow‐up (15.3%): 152 orders (8.6%) in 45 children (24.9%) had sub‐doses, while 117 orders (6.6%) in 62 children (34.3%) had overdoses. Among such wrong dose errors, 255 (94.8%) were disseminated throughout the medication system: 144 sub‐doses and 111 overdoses. Among the children with wrong dose errors, 58.1% had malnutrition versus 48.8% among those with no errors.

**Table 3:** Type of medication error by chemotherapy orders and patients

	Patients (n = 181)			Chemotherapy orders (n = 1762)		
Type of error	n	%	95% CI	n	%	95% CI
Prescribing	89	49.2	41.9‐56.5	278	15.8	14.1‐17.5
Wrong dose	86	47.5	40.2‐54.8	269	15.3	13.6‐16.9
Incomplete order	6	3.3	0.7‐5.9	9	0.5	0.2‐0.8
Transcribing^a^	6	3.3	0.7‐5.9	11	0.6	0.3‐1.0
Administration^b^	9	5.0	1.8‐8.1	9	0.5	0.2‐0.8

a Wrong doses.

b Includes only prednisone and dexamethasone omitted doses.

Regarding the magnitude of the wrong dose error, 17.7% of sub‐doses and 25.6% of overdoses had differences ranging from 25% to 50%, while 1.3% of sub‐doses and 11.1% of overdoses had differences greater than 50%. Among the latter, seven overdoses occurred in the prescription of corticosteroids and three with vincristine. However, a daunorubicin dose 8.5 times higher, caused by omission of a decimal point in the quantity ordered in the prescription, was the highest.

On the other hand, in the four transcription stages of the medication system, 11 orders (0.6%) with wrong doses were identified in the nurse's administration record, which is part of the clinical record (Table 3).

### 3.4 Consequences

In the four transcription stages, 11 MEs (3.7%) in chemotherapy orders with wrong doses did not reach the patient (Table 4): nine of them in the first transcription by the nurse and two appear corrected until the ECC records. Five intercepted MEs might have had consequences for the patient: two involved—12.1% and 49.3% differences in the vincristine dose that could have reduced treatment efficacy or increased chemotherapy toxicity (see Table 4 and Table S1).

**Table 4:** Medication error consequences

NCC MERP index categories	Patients^a^ (n = 104)			Chemotherapy orders (n = 298)		
	n	%	95% CI	n	%	95% CI
(B) Did not reach the patient	9	8.7	3.3‐14.1	11	3.7	1.6‐5.8
(C) Reached the patient with no harm	75	72.1	63.5‐80.7	262	87.9	84.2‐91.6
(E) Required treatment	10	9.6	3.9‐15.3	11	3.7	1.6‐5.8
(F) Prolonged or caused hospitalization	12	11.5	5.4‐17.7	12	4.0	1.8‐6.3
(I) Error may have contributed to death.	2	1.9	0.0‐4.6	2	0.7	0.0‐1.6

a The patients might be in more than one category: one patient appears in categories E and F due to different ADEs.

Of the 298 chemotherapy orders with any ME, 262 (87.9%) reached 75 children (72.1%) with no apparent consequences. However, 25 orders with MEs (8.4%) preceded ADEs in 23 children (Table 4). Among these orders, 16 had dosage deviations ranging from 10.1% to 25%, five were between 25.1% and 50%, and four were higher than 50%. Among the 23 patients affected by ADEs, 15 were girls (65.2%), 20 (87%) were classified at high risk and 13 (56.5%) had malnutrition.

In two deaths, MEs occurred that might have contributed to the process of death, one related to a cyclophosphamide overdose of 59.8% and another with pancreatitis onset after corticosteroid overdoses successive administration (a description of the cases is shown in Table S2).

### 3.5 Causes and preventability

**Figure 3:**
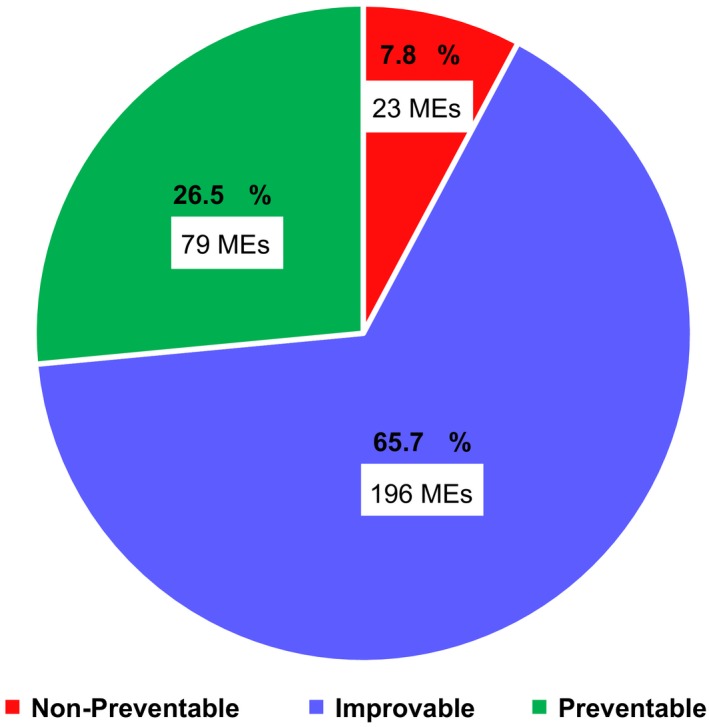
Preventability of medication errors. Preventable medication errors include those originating in the body surface area estimation and incomplete chemotherapy orders. Transcription and corticosteroid administration errors were included in the improvable group, as well as random calculation errors in the quantity of drug of the remission induction standard protocol. Wrong dose errors that originated in adaptations to the induction protocol or cytotoxic agents addition were not considered preventable

Two common causes for wrong dose error were inaccuracy in body surface area rounding, which occurred in up to 25.7% of the children's chemotherapy orders with wrong dose errors, and full dose calculations in patients who required reductions and that were not carried out in four children younger than 1 year or weighing less than 10 kg. Overall, only 23 MEs (7.8%) were judged as nonpreventable within our medication system; the rest originate from calculation processes that can be standardized and are highly likely to be improved (Figure 3).

## 4 DISCUSSION

This is the first study in a tertiary care hospital in Mexico to estimate the incidence of MEs in the pediatric oncology setting. MEs were common, but could be considerably reduced with preventive measures.^29^, ^30^, ^31^

In our medication system, based on handwritten prescriptions, an incidence of 57.5% of patients with MEs was observed. Other studies in Mexican tertiary care hospitals that use handwritten prescriptions have reported ME frequencies of 51%^30^ and 57.3%^32^ of patients in the general population and in the nononcological pediatrics setting, respectively. This suggests that institutions with handwritten prescription systems somehow share the magnitude of the ME problem. Other developing countries have reported MEs in 23.1% of children with ALL.^10^ Thus, these indicators of the medication process show the presence of a considerable, as well as highly preventable, problem.

Both the present study and the study conducted by Lavalle‐Villalobos^32^ highlight the dose calculation errors in pediatric care, which accounted for 90.2% and 35.1% of total MEs, respectively. In comparison, in the study by Hinojosa‐Amaya,^30^ wrong dose error is minimal (5.8%), with dose administration omission (68.5%) being the most common error in the general population. This difference reflects the complexity of weight or body surface area‐based dosing and the need to individualize or adjust very small doses by children age and growth.^33^, ^34^

However, the substantive appearance of MEs in the first phase of the medication process suggests that they could be reduced or more easily intercepted than in other stages.^35^ Preventive measures or interventions should put special emphasis on prescribing and dose calculation, especially to avoid those deviations greater than 25% that involved 26.7% of total wrong doses in this study, since systematic deviations can reduce the expected treatment efficacy or facilitate the appearance of ADEs related to excessive toxicity.^31^, ^36^ Said toxicity due overdose may have contributed more to mortality in our institution (0.7 per 100 MEs) than in other hospitals with surveillance systems (0.02%).^29^

In that sense, the proportion of intercepted MEs prior to administration to the patient was minimal: only 3.7% corresponded to wrong dose errors. This may be due to the fact that our medication process has multiple transcription stages and might be prioritizing the correct transmission of the prescribed dose rather than verifying its adequateness for the patient, thus facilitating the propagation of MEs. Other prospective studies with systematic safety and medication surveillance systems intercepted between 2% and 15.4% of prescribing errors.^29^, ^37^ Therefore, it is necessary to redesign our medication system in order for it to be fail‐safe.

The establishment of general measures for safe medication, such as personnel training programs, dose calculation verification by a second person (probably a nurse), body weight updating and application of standard criteria in body surface area rounding,^29^, ^31^, ^32^ as well as specific measures such as assistance of a clinical pharmacist^,^ 36, 37 and technological assistance systems^30^ are actions that have been shown to reduce the frequency of MEs,^30^, ^32^ and have the potential to increase patient safety.^31^

Although prescribing was the critical stage in the ME chain in this study, other studies in pediatric oncology identify the administration stage as the most important^7^, ^10^ Owing to the retrospective nature of this work, we consider it necessary to point out that the transcribing errors herein reported indicate that sometimes there were discrepancies between the dose documented by the nurse in the administration records and the chemotherapy dose prescribed by the attending physician and/or dispensed by the ECC when the latter was correct. Therefore, we were unable to distinguish between simple transcribing errors and serious errors related to the administration of wrong doses. For this reason, prospective studies are needed in order to delve into the role administration errors might play in oncological treatments at tertiary care hospitals.

MEs are known to be able to lead to ADEs. ADEs epidemiology varies widely between developing countries.^38^ A large study in North African countries reported the presence of adverse events in 8.2% of their records, where 34% were ADEs.^39^ In turn, the IBEAS study, which included five Latin American countries, reported 8.2% of ADEs.^40^ In this work, ADEs related to wrong doses were observed in 12.2 out of every 100 children under treatment, which is a higher number than that found in the two aforementioned large studies, and quite distant from the 1%‐2% rates observed in hospitalized cancer patients in developed countries.^38^ Although ALL induction therapy has the highest incidence of treatment‐related ADEs such as infections or thromboembolism, as patients receive chemotherapy for the first time while the primary disease is still active,^23^, ^41^ the ME rate at this stage is sufficiently high to allow assuming that the adoption of a medication system that is safer for the patient should lead to a reduction in the observed ADEs.

Finally, the data shown in this work can be useful in pediatric oncology tertiary care areas, where pharmaceutical surveillance systems for chemotherapy are incipient and technological resources and budgets are limited. These data are also an invitation to carry out studies to assess the safety of patient‐care systems.

## 5 LIMITATIONS

A strong limitation of this work was that 12.6% of the clinical records of children with newly diagnosed ALL could not be reviewed, either because they did not receive induction treatment at HIMFG (57.7%) or due to poor quality of the records (34.6%). The records of two patients could not be found, although they were searched for a few months later.

In addition, among the children included in the cohort, locating all the chemotherapy orders prescribed during induction treatment was not possible. Neither was it possible to compare the nursing administration records with the prescription and the ECC records in 1% of the reviewed orders, owing to the absence of such records. This shows that chemotherapy administration records have considerable room for improvement.

Another important limitation is the description of a short period of chemotherapeutic treatment of children with ALL in our hospital. This limits the applicability of our findings beyond induction, since at other stages, MEs and their causes can be quite different and, therefore, describing this phenomenon at subsequent phases of treatment is still necessary.

Finally, this study was not intended to evaluate the relevance of adaptations of the usual protocol based on physicians’ clinical judgment, but rather it assessed the quality of the process by means of which said decisions were carried out from prescription to administration. One example was the daunorubicin dose reduction to 75% (18.75 mg/m^2^) in patients assessed by the doctor as being at risk of complications if they received the full dose (25 mg/m^2^). In this case, the dose calculation was evaluated based on the new dosage proposed by the doctor and in consistency with the ECC preparation and nursing administration records.

## 6 CONCLUSIONS

In conclusion, MEs were common and occurred in 57.5% of children with ALL on induction treatment at HIMFG, as well as in 16.5% of chemotherapy orders. A considerable number of wrong dose errors (94.8%) propagated throughout the medication system, while 12.2 overdose‐related ADEs occurred for every 100 children on treatment. Such information should lead to the design of safer care processes, where individual actions of the health team take place in a fail‐safe environment, considering that only 3.7% of MEs generated in prescriptions were intercepted.

## CONFLICTS OF INTEREST

The authors declare not having any conflicts of interest.

## AUTHORS’ CONTRIBUTION

Edmundo Vázquez‐Cornejo was involved in conceptualization, investigation, methodology, formal analysis, validation, writing—review and editing. Olga Morales‐Ríos was involved in conceptualization, methodology, validation, writing—original draft, and writing—review and editing. Luis Enrique Juárez‐Villegas was involved in writing—review and editing. Erika Janet Islas Ortega was involved in data curation, writing—review and editing. Felipe Vázquez‐Estupiñán was involved in conceptualization, and writing—review and editing. Juan Garduño‐Espinosa was involved in conceptualization, project administration, methodology, and writing –review and editing.
