# Supplementary material for: Medication errors in a cohort of pediatric patients with acute lymphoblastic leukemia on remission induction therapy in a tertiary care hospital in Mexico
Source: Cancer Med. 2019 Aug 24;8(13):5979–87. doi: 10.1002/cam4.2438 (PMC6792484; doi:10.1002/cam4.2438)
Supplement: Supplementary file 1 [file CAM4-8-5979-s001.pdf]

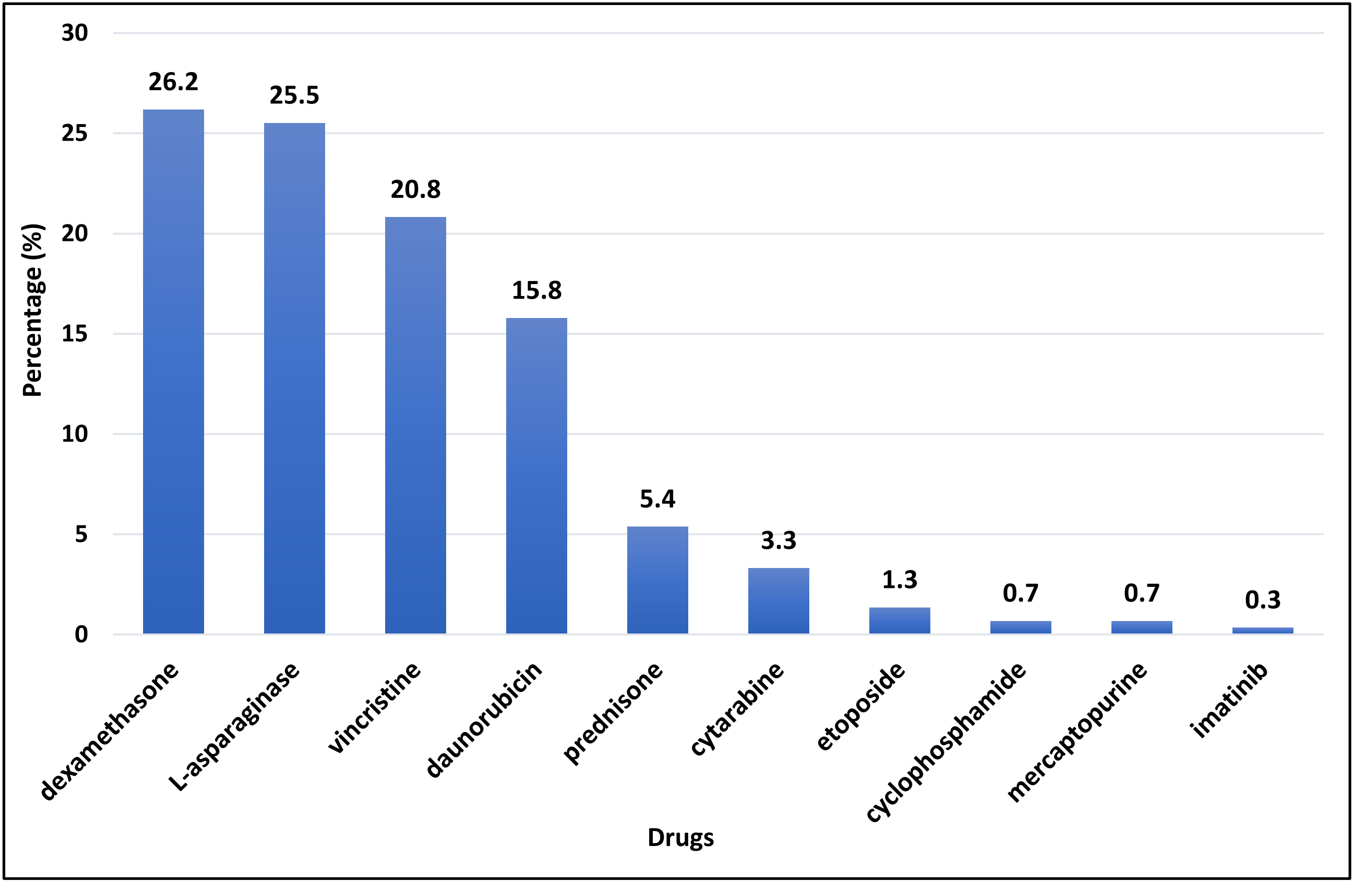

Supporting Figure 1. Proportion of medication errors per drug. The percentages shown reflect the fraction of medication errors for each chemotherapeutic drug. (n=298 medication errors).
