# Supplementary material for: Medication errors in a cohort of pediatric patients with acute lymphoblastic leukemia on remission induction therapy in a tertiary care hospital in Mexico
Source: Cancer Med. 2019 Aug 24;8(13):5979–87. doi: 10.1002/cam4.2438 (PMC6792484; doi:10.1002/cam4.2438)
Supplement: Supplementary file 2 [file CAM4-8-5979-s002.docx]

| **Supporting Table 1**. Potential adverse drug events related to intercepted wrong doses. | | |
| --- | --- | --- |
| Intercepted medication error | Clinical evolution | Potential adverse events (NCCMERP index) |
| **Potential adverse drug events related to wrong doses intercepted by the External Compounding Center.** | | |
| Vincristine:   - 49.3% overdose | 4-year 11-month-old girl with high-risk ALL and chronic undernutrition. On 03/16/2016, she developed neutropenic colitis and fever after the 03/12/2016 vincristine dose, which delayed induction for 12 days.  After 42 days of induction treatment (32 as inpatient), she experienced clinical symptoms of vincristine-associated ototoxicity. | - Might have prolonged hospital stay and treatment delay due to neutropenic colitis and fever. (F) - Potential ototoxicity increase, as a permanent damage. (H) |
| Vincristine:   - 12.1% sub-dose44627 | One-year 3-month boy with standard-risk ALL, who developed induction failure at day 14 (12/31/2017). | - Could have contributed to induction failure. (E) |
| **Potential adverse drug events related to wrong doses intercepted by the nurse** | | |
| Dexamethasone:   - 96.7% overdose - 32.1% overdose - -18.9% sub-dose | Six-year-old boy with standard-risk ALL, stable at admission and who showed signs and symptoms of sepsis at the moment of prescription.  Twelve-year adolescent, high-risk ALL, hemorrhagic syndrome at admission and overweight.  Two-year 7-month boy with high-risk ALL, clinically complicated at admission: he presented with hemorrhagic syndrome, tumor lysis and multiple previous infectious processes. | Risk of hyperglycemia or other corticosteroid-related adverse events or infections. (E)  Risk of hyperglycemia or other corticosteroid-related adverse events or infections. (E)  Risk of poor response to corticosteroid window at day 8. (E) |
| Other 6 dosing errors were intercepted: 2 with vincristine with differences of 10.1%, 2 with dexamethasone with differences of 10.4% and 11.5%, and with etoposide and cytarabine with differences of 8.8%, but with 32-mg correction; these errors were considered potentially without consequences (C). | | |
