# Supplementary material for: Medication errors in a cohort of pediatric patients with acute lymphoblastic leukemia on remission induction therapy in a tertiary care hospital in Mexico
Source: Cancer Med. 2019 Aug 24;8(13):5979–87. doi: 10.1002/cam4.2438 (PMC6792484; doi:10.1002/cam4.2438)
Supplement: Supplementary file 3 [file CAM4-8-5979-s003.docx]

| **Supporting Table 2**. Deaths where medication errors occurred. | | |  |
| --- | --- | --- | --- |
| Medication error | Clinical evolution | Case evaluation | Suggested preventive measure |
| **Case 1** | | | |
| Cyclophosphamide  59.8% overdose:  Instead of 250 mg, 400 mg were prescribed. | 5-year-old boy with standard-risk ALL and chronic undernutrition who experienced multiple gastrointestinal adverse events and septic shock with deep immunosuppression after remission-induction treatment. A fifth cytotoxic agent was added in order to intensify the treatment due to poor response at day 14, but he died a few hours after cyclophosphamide administration. | Although, the patient was severely ill, overdose was ongoing when death occurred.  It may have contributed to patient death along with the rest of complications secondary to the treatment and to ALL. | Dose calculation double-check by a second person. |
| **Case 2** | | | |
| Prednisone  16.8% overdose  20 mg orally every 8 h were prescribed instead of 17 mg every 8 h. | Ten-year-old boy with high-risk type B ALL and overweight, who was admitted to the hospital in serious conditions with bleeding and tumor lysis syndrome. Corticosteroid window with prednisone overdose was started; he received it for 4 days; on the fifth day, there were clinical complications, and acute pancreatitis secondary to corticosteroids was diagnosed, after which prednisone was changed to dexamethasone.  However, the patient died within the ensuing 24 hours due to complications secondary to the adverse drug event and septic shock with multiple organ failure. | The relationship between high-dose corticosteroid administration and pancreatitis is possible according to the literature and there was clinical diagnosis during hospitalization and autopsy pathological confirmation. However, it is a rare complication.**^1^**  The administration of high-dose corticosteroid at the beginning of induction is part of the treatment, and it is not clear if the absence of the prednisone overdose would have prevented the onset of pancreatitis. However, the medication error appears in the chain of events that culminated in the boy’s death. | Dose calculation double-check by a second person.  Ensure adequate corticosteroid dose in children who have a delicate clinical status. |
| **^1^**Adverse Effects: Prednisone (electronic version). IBM Watson Health information, https://www-micromedexsolutions-com.pbidi.unam.mx:2443/. Accessed on March 28, 2019. | | | |
